# Effects of Individual Mortality Experience on Out-of-Wedlock Fertility in Eighteenth- and Nineteenth-Century Krummhörn, Germany

**DOI:** 10.1007/s12110-020-09368-3

**Published:** 2020-06-16

**Authors:** Katharina E. Pink, Kai P. Willführ, Eckart Voland, Paul Puschmann

**Affiliations:** 1 Family and Population Studies, Centre of Sociological Research, KU Leuven, Parkstraat 45, 3000 Leuven, Belgium; 2 Department of Evolutionary Anthropology, University of Vienna, Althanstrasse 14, 1090 Vienna, Austria; 3 Department I Educational- and Social Sciences, Carl von Ossietzky University of Oldenburg, Postfach 2503, 26111 Oldenburg, Germany; 4 Institute of Philosophy, Justus Liebig University Gießen, Rathenaustraße 8, 35390 Gießen, Germany; 5 Radboud Group for Historical Demography and Family History, Radboud University, Erasmusplein 1, 6525 Nijmegen, The Netherlands

**Keywords:** Life history theory, Reproductive timing, Illegitimacy, Mortality, Krummhörn

## Abstract

Life history theory predicts that exposure to high mortality in early childhood leads to faster and riskier reproductive strategies. Individuals who grew up in a high mortality regime will not overly wait until they find a suitable partner and form a stable union because premature death would prevent them from reproducing. Cox proportional hazard models were used to determine whether women who experienced sibling death during early childhood (0–5 years) reproduced earlier and were at an increased risk of giving birth to an illegitimate child, with illegitimacy serving as a proxy for risky sexual behavior. Furthermore, we investigate whether giving birth out of wedlock is influenced by individual mortality experience or by more promiscuous sexual behavior that is clustered in certain families. Models are fitted on pedigree data from the eighteenth- and nineteenth-century Krummhörn population in Germany. The results show a relationship between sibling death in early childhood and the risk of reproducing out of wedlock, and reproductive timing. The risk of giving birth out of wedlock is linked to individual mortality experience rather than to family-level effects. In contrast, adjustments in connubial reproductive timing are influenced more by family-level effects than by individual mortality experience.

**Electronic supplementary material:**

The online version of this article (10.1007/s12110-020-09368-3) contains supplementary material, which is available to authorized users.

Until the mid-twentieth century, childbearing in most European countries was strongly linked with marriage, which marked the onset of sexual reproduction. In western Europe, marriage occurred well after biological maturation due to culturally imposed norms such as the economic self-sufficiency of newlyweds (Engelen and Wolf 2005; Hajnal 1965). Although there was a considerable time-lag between physical maturation and age at first marriage, out-of-wedlock fertility was rather low in premodern times because giving birth to an illegitimate child was associated with negative consequences for both the mother and the child (Laslett et al. 1980; Mitterauer 1983). Besides the social stigma, fathers of illegitimate children were often absent, mostly invested few or no resources in their children’s upbringing, and infant mortality was higher than among legitimate children (Gardarsdóttir 2000). Furthermore, previous studies linked father absence in early childhood to the reproductive development (e.g., age at menarche, age at first sexual intercourse) of the children (e.g., Draper and Harpending 1982; Ellis et al. 2003; Matchock and Susman 2006; Mendle et al. 2009). A recent review by Sear et al. (2019), however, showed no support for a universal acceleration of puberty in households where the father was absent. In general, giving birth out of wedlock was a risky reproductive strategy in the historical context.

Roughly between the end of the eighteenth century and the middle of the nineteenth century, European countries experienced a considerable and, in some cases, even a dramatic increase in illegitimate births. The phenomenon was mainly found among the working classes, and although certain cities, such as Stockholm and Vienna, reached very high levels of out-of-wedlock fertility, many rural areas were also affected. Several potential explanations have been put forward in the literature for the rise in illegitimacy. Shorter (1975) interpreted it as the result of an early sexual revolution, whereas Tilly et al. (1976) argued that men more often broke marriage promises because of economic challenges posed by early industrialization and urbanization. Laslett (1980) found that many of the women who gave birth to children out of wedlock were linked to each other and were “repeaters”—they gave birth to multiple illegitimate children, who were themselves at an increased risk of becoming the parents of illegitimate children. He suggested that these women and men were part of a bastardy-prone group and stated that within this subpopulation, social norms regarding sexuality and marriage deviated from those of the rest of society. Kok (2005), by contrast, saw the rise in out-of-wedlock fertility as a consequence of the breakdown of social control systems in the wake of urbanization and industrialization.

Although numerous scholars have attempted to explain out-of-wedlock fertility from a historical perspective, taking the agency of historical actors as well as changing contexts into account, sociobiological perspectives are pretty much missing. In this regard, little is known about the impact of exposure to high mortality during earlier childhood on the risk of giving birth out of wedlock with the exception of Scott and Duncan’s (1997) observation of a link between price hikes in wheat, adult mortality, migration, and a rise of illegitimate births in preindustrial northern England.

Life history theory predicts that mortality experiences in early childhood lead to faster and riskier life strategies, and such mortality experiences have indeed been shown to lower the age at first birth in both preindustrial and contemporary societies (Nettle et al. 2011; Placek and Quinlan 2012; Quinlan 2010; Störmer 2011). There are, accordingly, good reasons to test whether life history theory predictions also hold in the case of culturally imposed restrictions of reproduction to marriage. Does exposure to high mortality lead to a higher risk of engaging in premarital intercourse, resulting in an increased risk of giving birth to an illegitimate child in the population under observation? Can individual mortality experience within the natal family during early childhood add a further explanation to out-of-wedlock fertility?

## A Life History Approach to Out-of-Wedlock Fertility

During its life cycle an organism harvests energy from the environment and makes certain trade-off decisions regarding the investment of resources in terms of age-specific functions such as growth, maintenance, and reproduction (Roff 2002; Stearns 1992). Life history theory posits that these trade-off decisions are influenced by local ecological circumstances, and natural selection favors life history strategies that optimize resource allocation over an individual’s life course (Kirkwood 1977; Schaffer 1983; Williams 1957). The level of extrinsic mortality is one important ecological factor that influences the timing of certain life events (Kirkwood and Rose 1991; Promislow and Harvey 1990). Extrinsic mortality is a result of environmental hazards such as infectious diseases, predation, war, famine, or accidents and individuals cannot escape or control it by behavioral change (Gurven and Fenelon 2009; Quinlan 2007). It manifests as environmental harshness or environmental unpredictability (Ellis et al. 2009). Environmental harshness varies spatiotemporally and is largely inevitable. Environmental unpredictability is the random variation of environmental harshness under which the success of behavioral adaptation is arbitrary (Kavanagh and Kahl 2016). In unpredictable environments, long-term investments might not pay off due to premature death. High uncertainty results in devaluating the future; hence individuals tend to shift their time perspective from future-oriented to present-oriented (Brumbach et al. 2009) and adapt their behavior accordingly (Adams and Nettle 2009): Life is here and now. Present-oriented individuals devaluate the future and pursue high-risk strategies favoring immediate and short-term gains (Hill et al. 1997, 2008). They invest less in growth and maintenance and shift their resources toward early maturation and reproduction. A faster life history strategy with an early onset of reproduction to obtain an optimal fitness maximization strategy could be seen as an adaptive strategy to beat the odds of not reproducing at all in a harsh and unpredictable environment where life expectancy is reduced (Anderson 2010; Griskevicius et al. 2011; Low et al. 2008; Wilson and Daly 1997). Previous studies in contemporary societies have shown that exposure to high mortality during early childhood (0–7 years) can trigger fast life history strategies characterized by earlier age of menarche, first pregnancy, and birth (Belsky et al. 2010; Chisholm et al. 2005; Nettle 2010; Nettle et al. 2011; Quinlan 2010). Furthermore, it is well documented that earlier age of menarche is strongly associated with earlier age at first date, first kiss, and risky sexual behavior such as earlier age at first sexual intercourse (Hoier 2003; Lam et al. 2002) and a higher prevalence of teenage pregnancies (Nettle et al. 2011; Romans et al. 2003). The early onset of reproduction in turn entails risks such as higher neonatal and infant mortality, and a higher likelihood of giving birth to low-birth-weight, premature, and small-for-gestational-age infants (Fraser et al. 1995; Olausson et al. 1999). Despite these risks, in high-mortality environments an accelerated life strategy (faster maturation and early reproduction) reduces the risk of death prior to producing offspring (Brumbach et al. 2009).

### Objectives

The present study uses preindustrial historical longitudinal data from the Krummhörn region in northwest Germany from the eighteenth and nineteenth centuries to study the effects of high mortality as measured by exposure to sibling death on young women’s risk of bearing an illegitimate child. The rearing environment is an important point of reference for future environmental conditions an individual might have to face. Focusing on exposure to sibling death instead of measuring mortality on a higher level of aggregation allows us to investigate the influence of the family environment on the development of an individual’s life history strategy. Siblings are not necessarily exposed to the same environmental cues because of the timing of certain events and the individual’s birth order. Störmer and Lummaa (2014) pointed out the importance of family environment rather than individual mortality experience. They hypothesize that some kind of “family mentality” is at play: parental behavior is stress-sensitive, and loss of a child will have an impact on it. In line with Chisholm (1993), they argue that the surviving offspring are confronted with an unpredictable environment and therefore adapt their life history strategies accordingly. Furthermore, we address whether shared family environment, such as genetic predisposition (Barban et al. 2016), and heritability of fertility traits, such as age at first reproduction (Bolund et al. 2013; Milot et al. 2011) or individual mortality experience within the natal family, trigger riskier and faster life histories of women.

## Data and Methods

### Study Population and Period

Our data are derived from a family reconstitution dataset based on Protestant church registers and tax rolls of the Krummhörn region in East Frisia (Germany) from the eighteenth and nineteenth centuries (for a comprehensive description of the database and the methodology see Voland 2000). Historical Krummhörn was divided into 33 neighboring parishes, all of which are included in the dataset. The dataset contains 118,778 individuals who were in 34,708 marriages. It is archived at the GESIS-Institute (Cologne).^1^ Many of the records dated before 1720 are incomplete, and families from the social and economic elite tend to be overrepresented in these early records. After 1874, the church was no longer responsible for maintaining records of births, deaths, and marriages, as this task had been transferred to the civil registry offices (Standesämter). Due to the bias in the early records and the censoring after 1874, we limit our analysis to females who were born between 1720 and 1850 (*N* = 25,487) and do not include events after 31 December 1874 in our analyses. From this sample, 11,874 women are censored before possible childbirth since they either migrated out of the study area unmarried or did not become mothers before 1874. Our final sample contains 8339 women who gave birth to their first child within marriage and 379 women who gave birth to their first child out of wedlock and never married the father of this child. Five of these 379 women were born out of wedlock themselves and were never legitimized. We run two different models: one excluding these five women and the other including them as well as the information about their context of origin via a dummy variable. Since the model effects of the model excluding those women were quite similar to results given in A1 and A2, we proceed with the model that includes the women who were born illegitimate themselves.

Geographically, the peripheral rural region of the Krummhörn is bordered to the north and west by the North Sea, to the south by the River Ems, and to the east by sandy soil and moorlands, which were impenetrable at that time. The Krummhörn region itself had very fertile marsh soil that was suitable for raising both crops and livestock. The Krummhörn region had been fully settled by the late medieval period (Ohling 1963), and there was no significant population growth during the study period (Klöpper 1949). Since the region was a saturated habitat with a finite amount of arable land, the population faced local resource competition (Voland and Dunbar 1995). Due to the limited access to land, a stratified social structure arose among the Krummhörn population. The large-scale farmers with capital and status were at the top of this social hierarchy, and the small-scale farmers, craftsmen, and landless workers occupied the lower end of this social structure. In the eighteenth century, about 70% of the Krummhörn’s families either had no land at all or their farms were too small to ensure subsistence, and thus they were forced to supplement their income by working for the large-scale farmers (Willführ and Störmer 2015). Although there are no records indicating that the region was affected by famine or war during the study period, smallpox and other infectious diseases took a significant toll on the people of the region, as in other parts of Europe, over the course of the eighteenth century (Omran 2005). The average family size was about four children (Voland and Dunbar 1995; Willführ and Störmer 2015). The families of the region practiced a form of ultimogeniture in which the youngest son inherited the undivided farm from the father and the other offspring had to be compensated, often with cash (Ohling 1963). A daughter could expect to receive half of the amount of compensation each son received. Due to these inheritance practices, families in the Krummhörn region tended to be relatively small and the average age at first marriage was high (female average age at first marriage: 26.285 years (SD = ± 5.406; Willführ and Störmer 2015). Thus, late reproduction and low birth rates were the norm.

### Modeling the Risk to Give Birth out of Wedlock

We use Cox regression (Allison 2014; Cox 1972) to model the life course of reproductive women, starting at birth and continuing to the age of first childbirth. “Reproductive women” in this context means that these women gave birth at least once in their lifetime regardless of whether the children were born within marriages or out of wedlock. As mentioned above, we use the event of giving birth out of wedlock as a proxy for increased risk-taking behavior. The traditional definition of giving birth out of wedlock refers to all extramarital births (Laslett et al. 1980). This definition, however, might fall short in the case of the Krummhörn region because it was not uncommon for couples to marry after conception or shortly after the birth of their first child. This reproductive behavior may not reflect increased risk-taking, but structural factors such as marriage bans during the harvest season or during religious holidays. We therefore stick to a strict definition: “Born out of wedlock” means that the woman never married.

In estimating the effects of sibling mortality experience, we rely on a combination of models adjusted for clustering at the family level, and models stratified at the maternal level (family fixed effects; see Allison 2009). The former models investigate the general association between mortality experience and the risk of giving birth out of wedlock. The latter models estimate likelihood functions with separate terms for each of the families in the dataset and thus allow each family to have their own individual baseline hazard function. The key difference between the stratified and the clustered Cox regression models is that the stratified models identify the effect of mortality experience using the variation within families, but not between families. These stratified models control for unobserved heterogeneity—for example, genetic and environmental factors if these were shared by sisters. By comparing the results of the clustered models with the results of the stratified models we try to assess whether the risk of giving birth out of wedlock is affected by individual mortality exposure or whether mortality and more promiscuous sexual behavior is clustered in certain families. The flaw of models that are stratified on the family level is that singlets (reproductive women without reproductive sisters) are excluded from the analysis. Therefore, if the results of a clustered model version are not in line with the results of the corresponding stratified version, one needs to check if the different results are due to different sample sizes or to differences in the hazard function estimation. A similar approach was used by Fox et al. (2017) to study whether having siblings affects mortality and reproductive success and by Willführ et al. (2018) to study kin effects on the mortality of reproductive women.

The time-varying information about early experience of sibling death(s) is included as a dummy variable in the models. We only consider deaths of siblings that occurred during the childhood of the individuals of interest. Sibling death(s) before the individual of interest’s birth or after their fifteenth birthday are not considered in the analysis. To investigate whether a sibling mortality experience effect is age-specific, we varied the age range in which we consider sibling deaths. Life history theory and the theory of evolutionary socialization predict that mortality experience is not equally effective over the juvenile period. We expect to find “sensitive windows” where the loss of siblings is most effective. Therefore, we decided to employ a model in which sibling death experience before the age of 15 is coded in three variables: sibling death experience (a) between birth and the age of 5, (b) between the ages of 5 and 10, and between the ages of 10 and 15. We ran different models to vary the chosen age range. The results of these models are available online in the Electronic Supplemental Material (ESM). In order to control for potential confounders, we include time-varying information about the number of siblings alive (as a measurement for current family size) and whether the father or mother of the individual was deceased. Each change in one of these covariates is an event, which brings a new episode of observation to the model.

We further include birth order, birth cohort, and the parental socioeconomic status (SES) as time-invariant control variables in the models. The possibility of experiencing sibling death is dependent on the individual’s birth order and on sibling cohort size since, for example, firstborns are not exposed to sibling mortality until the parents have a second child. Female birth cohort is coded in decades and is included as control for time trends in fertility and mortality. All cases are categorized into five groups based on their parents’ land ownership status. Families who owned more than 75 *Grasen* (an old German areal measure, 1 Gras ~0.37 ha) are classified as “large-scale farmers”; families who owned between 10 and 75 Grasen are assigned to the “mid-scale farmers” group, and families who owned less than 10 Grasen are categorized as “small-scale farmers.” Families who had no land are classified as “landless,” and families for whom the level of land ownership was unknown are placed in the “unknown” group. The borders between these categories are more or less arbitrary but fit well into the historical context (Beise 2001; Willführ and Störmer 2015). All analyses were performed in R-3.5.3 along with the following packages: data.table, reshape, and Hmisc (includes survival), broom, and ggplot2.

### Limitations

Because of the substantial migration of young adults out of the study area, the death dates of many individuals are missing. This might pose a problem for the current study design. We can assume that children survived to adulthood if the parents’ marriage was under observation. This criterion is fulfilled if the start and end dates of the marriage are recorded, and we only include individuals who derived from such families. We therefore can assume survival of siblings up to the age of 15 if their date of death is missing. This procedure might result in a systematic underestimation of sibling death. However, infant and child survival estimates based on this selection criterion are in line with estimates based on census data. We believe that this potential underestimation is not interfering with our research questions since we might face the problem only of false negative results, but not of false positive.

## Results

**Fig. 1:**
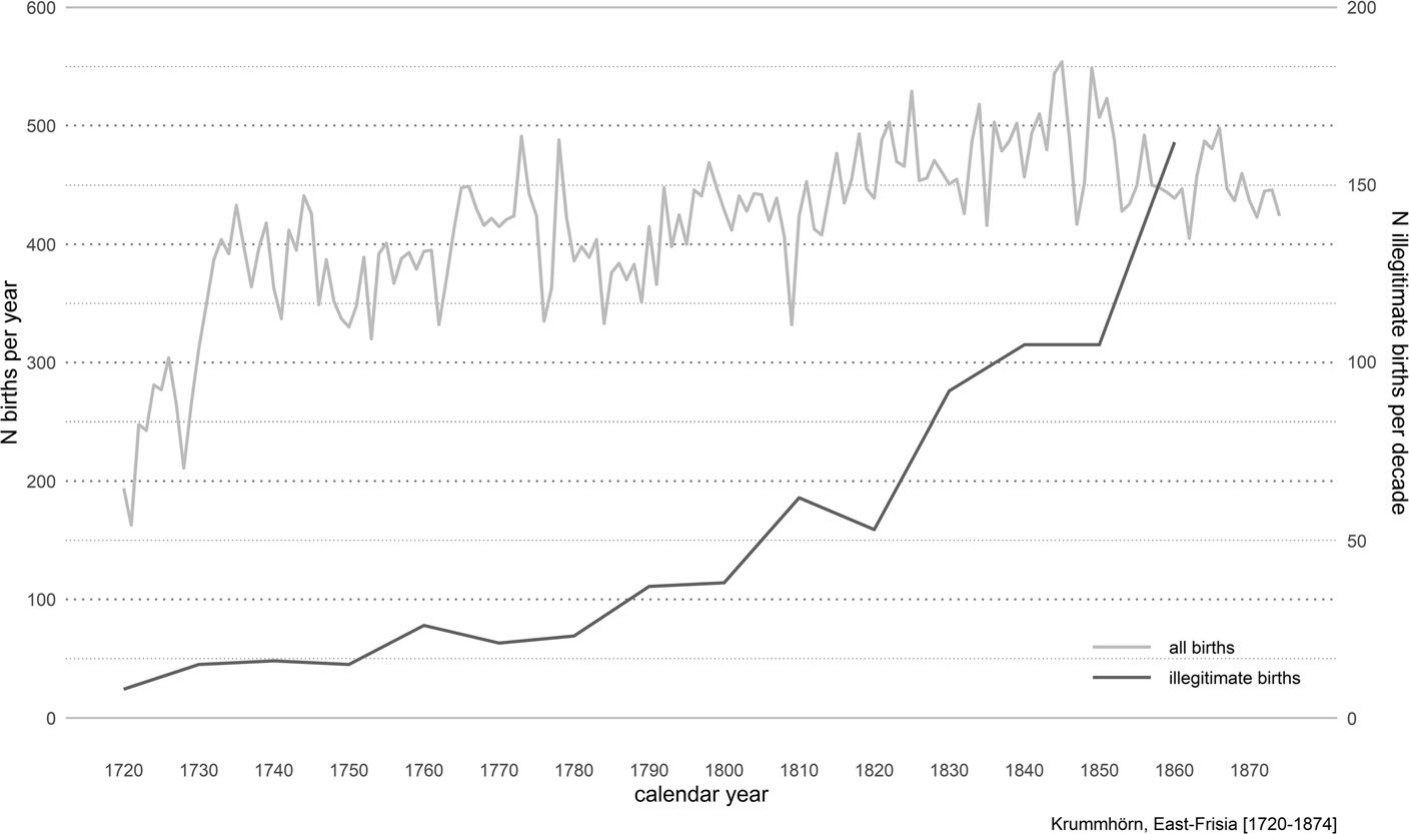
Number of births and proportion of illegitimate births per calendar year for the Krummhörn population (Ostfriesland, Germany)

Consistent with previous literature (e.g., Laslett et al. 1980; Mitterauer 1983; Shorter 1975), we find that the proportion of illegitimate births started to increase at end of the eighteenth century (Fig. 1). This trend was consistent through the end of the study period in 1874.

**Fig. 2:**
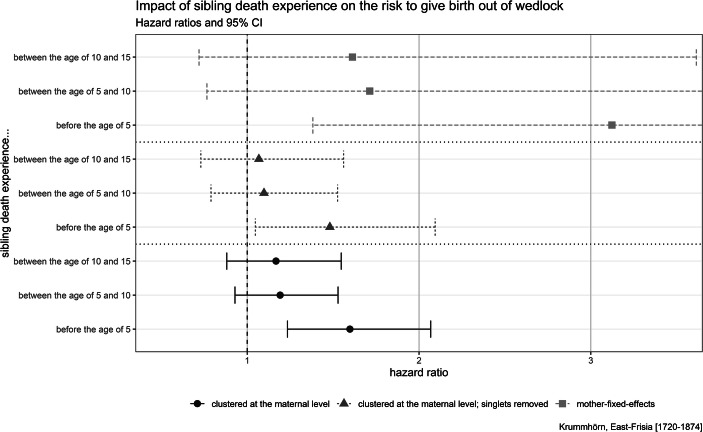
Results of the Cox regression modeling the risk of giving birth out of wedlock. Models control for number of siblings alive, whether father or mother has died, child’s birth cohort (in decades), child’s birth order, and parent’s socioeconomic status (omitted in the mother-fixed-effect versions). Results of the full models, including tests for proportional hazard assumption, are given in A1 in the ESM

The results of the Cox regression, which estimate the effect of sibling mortality experience on the risk of giving birth out of wedlock, are summarized in Fig. 2. The full models including test for proportional hazard assumption are given in A1 in the ESM. We find that sibling death experience before the age of 5 is statistically significantly associated with an increased risk of giving birth out of wedlock. The impact of sibling death experience before the age of 5 is detectable in the clustered as well as the fixed-effect model versions, which indicates that this association is driven by individual experience and is not due to unobserved shared family characteristics, such as genes and environmental factors. Sibling death experience between the ages of 5 and 10 as well as between 10 and 15 does not significantly affect the risk of giving birth out of wedlock.

**Fig. 3:**
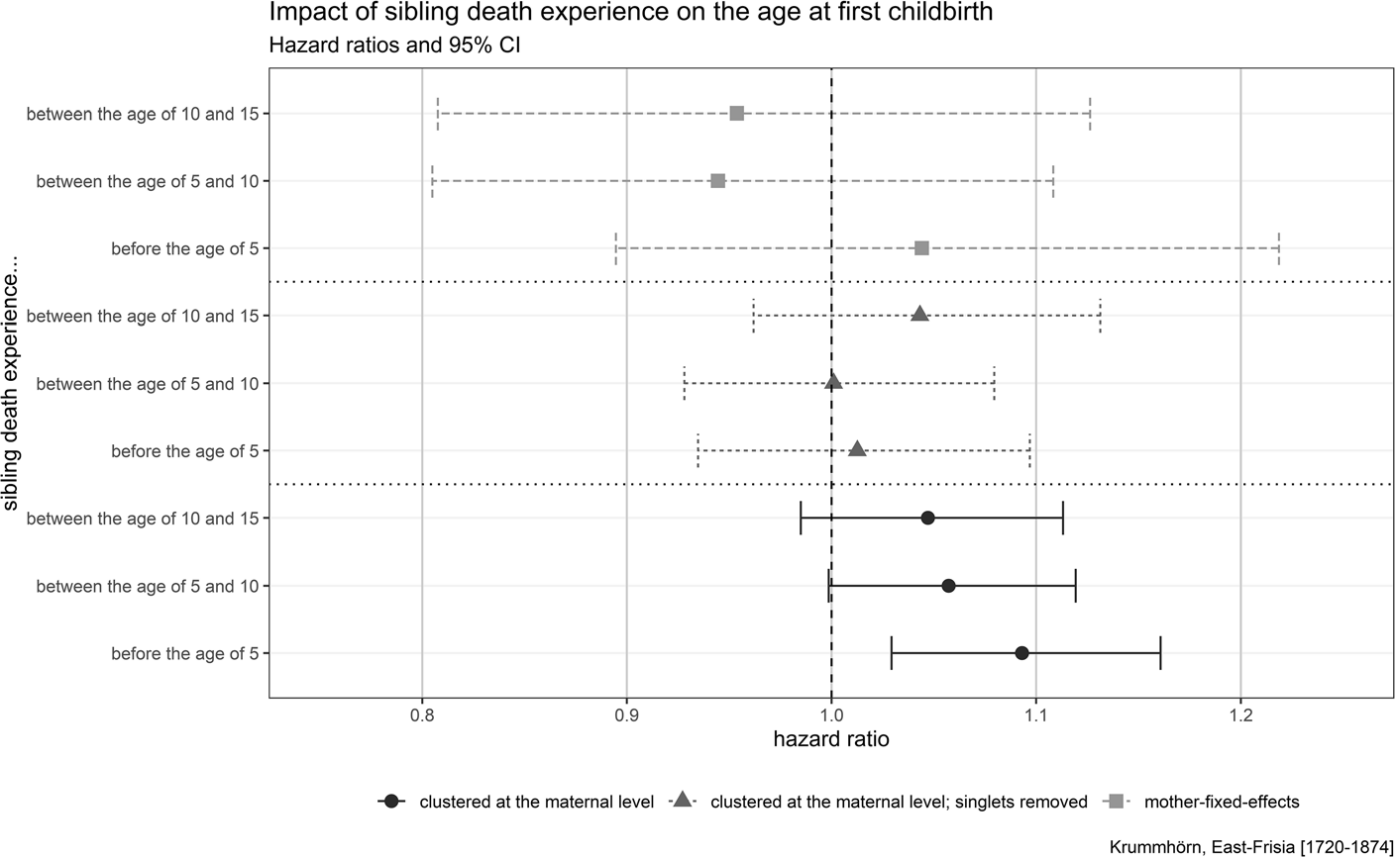
Results of the Cox regression modeling time to first childbirth. Models control for number of siblings alive, whether father or mother has died, child’s birth cohort (in decades), child’s birth order, and parent’s socioeconomic status (omitted in the mother-fixed-effect versions). Results of the full models, including tests for proportional hazard assumption, are given in the A2 in the ESM

The results of the Cox regression, which estimate the effect of sibling mortality experience on timing of first childbirth, are given in Fig. 3. The full models, including tests for proportional hazard assumption, are given in A2 in the ESM. Like the model estimating the risk of giving birth out of wedlock, the clustered model version estimating the time to first childbirth suggests that sibling death experience before the age of 5 is statistically significantly associated with earlier childbirth. The association, however, is found neither in the fixed-effect model nor by the clustered model version that excludes singlets (reproductive females without a reproductive sister in the data). This is an indication that the association is explained by unobserved shared family characteristics or by factors that are absent in single-daughter families.

To test whether the association between sibling death experience in childhood and the risk of giving birth out of wedlock may be driven by confounding factors, we ran several robustness checks (presented in the ESM). First, we investigate whether the effects are explained by the number or incidence of sibling death experiences. In A3 we (re-)transform the dichotomous variables coding for the number of deceased siblings. The results of the models with these modified variables are comparable with the models in Fig. 2. Second, we are interested in the raw effects of the variables of interest (estimated in models without controls; see A4). Third, we run two different models to verify the chosen age range between birth and the age of 5. In 0.1-year increments we extend the age range in which we include the information about sibling death(s). In the model series presented in A5 we start from birth until the first month and then extend the age range stepwise until we cover the range from birth up the age of 15. In the model series presented in A6 we start from age 15 and extend the age range in the same manner down to birth. The analysis suggests that the chosen age range between birth and the age of 5 is justified: Models which exclude this range do not suggest statistically significant associations.

## Discussion

Most studies put great emphasis on the importance of individual and historical context— industrialization, urbanization, and their economic and social effects—in explaining out-of-wedlock fertility in Europe in the eighteenth and nineteenth centuries (Mitterauer 1983; Schumacher et al. 2007). Our study incorporates life history theory as an additional explanatory factor. We assume that individuals experiencing high mortality (via sibling death) are discounting the future, favor immediate rewards over delayed rewards, and pursue risky reproductive strategies. “Risk” in this context means that they give birth at an earlier age and have more illegitimate children because they start having sex before a stable relationship is established and a marriage tie is formalized. In line with previous research (Störmer and Lummaa 2014; Voland and Willführ 2017) we distinguish between the impact of individual mortality experiences within the natal family and family-level effects (shared environment/genetic predisposition). Although child mortality was generally decreasing in the period under observation, the results obtained in this study stressed the importance of the individual mortality experience during early childhood on an individual’s life trajectory. Women who witnessed the death of one or more of their siblings in early childhood (0–5 years) were more likely to give birth out of wedlock. The environmental conditions in the first 5 years of life are particularly important since humans are primed to learn essential lessons regarding pair-bonding and child-rearing behavior during that time (Belsky et al. 1991). Witnessing a sibling’s death during this sensitive window conveys the impression of ecological unpredictability, which has been associated with increased future discounting and risk taking (Hill et al. 1997; Lee et al. 2018). A present-oriented individual is devaluating the future and therefore is assumed to invest more in riskier behavior that might result in immediate rewards (e.g., all-in investment in the current relationship, including premarital sexual intercourse) than in future-oriented behaviors that require long-term planning and investments (e.g., courtship, establish a stable union and an independent household; Boyd and Zimbardo 2005; Schechter and Francis 2010). Even when individuals have to cope with resource uncertainty, high child mortality, and the risk of being stigmatized and socially excluded (Brändström 1996; Gardarsdóttir 2000; Kok et al. 1997; Laslett et al. 1980), the immediate reward of reduced risk aversion and therefore diminished self-control might outweigh such drawbacks. In contrast to findings in other populations, giving birth to an illegitimate child in the Krummhörn population cannot be seen as an alternative reproductive strategy. In the population under observation, women invested everything in the current relationship, including premarital sex. Such risky sexual behavior could pave the way for marriage and stable family formation, but it could also lead to a precarious situation in which pregnant women were abandoned. Our results clearly show the importance of the individual’s early-life mortality experience (i.e., the number of siblings who died is related to the tendency to give birth out of wedlock) in pursuing a riskier reproductive strategy.

Apart from showing the tendency to reproduce out of wedlock, our results also indicate that women who experienced the deaths of siblings during early childhood (i.e., 0–5 years) started reproduction earlier. In contrast to the results on out-of-wedlock fertility, family membership is revealed to be more important than the individual mortality experience with respect to timing of first birth. This is in line with previous research: using data from three different historical populations (Finland, Quebec, and Krummhörn), Störmer and Lummaa (2014) demonstrated that the family environment is more important for modifying reproductive timing than the individual mortality experience within the natal family. It is assumed that parents respond to the loss of a child in a high-mortality environment by reducing their per-capita investment in surviving offspring (shift in the quality-quantity-trade-off), thereby creating an uncertain environment for the surviving offspring, who themselves respond by adapting their life histories (Belsky et al. 1991; Chisholm 1993).

We also examine the time-dependent effect of exposure to sibling deaths with interactions on the likelihood of giving birth to an illegitimate child and age at first birth. Our results show that mortality exposure is interacting with time, more precisely with the stage of ontogenetic development, in the Krummhörn population. Thus, mortality exposure in early life affects reproductive timing and decision-making to a greater degree than in later phases of childhood. It has already been demonstrated that children who experience high mortality tend to develop insecure attachment styles (Chisholm 1993). Insecure attachment is associated with faster development, looser bonds, present-oriented and riskier behavior, low parental effort, and low mating effort (Ivan and Bereczkei 2006; O’Connor et al. 1999; Schechter and Francis 2010).

In sum, a growing body of research suggests an important role of mortality in shaping human reproductive strategies. The current study indicates that mortality in the form of exposure to sibling death during early childhood is significantly associated with the risk of giving birth out of wedlock in the eighteenth- and nineteenth-century Krummhörn population. It underlines the importance of life history theory in explaining reproductive behavior of individuals in general and the timing of the onset of reproduction as well as the likelihood of giving birth out of wedlock in particular.

## Electronic supplementary material

ESM 1 (PDF 262 kb)

## Footnotes

1 The data are available from the dataset maintainer GESIS-Institute (Cologne) with the label ZA8630 (10.4232/1.12643).
