# Supplementary material for: Effects of Individual Mortality Experience on Out-of-Wedlock Fertility in Eighteenth- and Nineteenth-Century Krummhörn, Germany
Source: Hum Nat. 2020 Jun 16;31(2):141–54. doi: 10.1007/s12110-020-09368-3 (PMC7381461; doi:10.1007/s12110-020-09368-3)

Electronic Supplementary Material (ESM) for

Effects of Individual Mortality Experience on Out-of-Wedlock Fertility in Eighteenth- and Nineteenth-Century Krummhörn, Germany

Katharina E. Pink*, Kai P. Willführ, Eckart Voland, Paul Puschmann

Human Nature 31(2), 2020, https://doi.org/10.1007/s12110-020-09368-3

*corresponding author: katharina.pink@kuleuven.be

Family and Population Studies, Centre of Sociological Research, KU Leuven, Parkstraat 45, 3000 Leuven, Belgium

Department of Evolutionary Anthropology, University of Vienna, Althanstrasse 14, 1090 Vienna, Austria

A1-Full models for figure 2

```
Call:
coxph(formula = Surv(start, end, dummy) ~ sibdead5 + sibdead5_10 +
    sibdead10_15 + famsize + dadD + momD + cohort + birth rank +
    socrank, data = sample, method = "efron", cluster = idf)

  n= 75902, number of events= 379

                     coef exp(coef) se(coef) robust se     z Pr(>|z|)
sibdead5          0.461497  1.586447 0.125305  0.131416 3.512 0.000445 ***
sibdead5_10       0.167975  1.182907 0.126961  0.126837 1.324 0.185391
sibdead10_15      0.162196  1.176091 0.137046  0.142559 1.138 0.255228
famsize           0.084818  1.088519 0.025807  0.026671 3.180 0.001472 **
dadD              0.187404  1.206114 0.110842  0.120033 1.561 0.118460
momD              0.106220  1.112067 0.121478  0.129631 0.819 0.412556
cohort            0.019654  1.019848 0.002240  0.002481 7.922 2.34e-15 ***
birth rank        0.014512  1.014618 0.026060  0.026972 0.538 0.590550
socrank2          0.393138  1.481623 0.605929  0.653053 0.602 0.547174
socrank3          0.487240  1.627817 0.585943  0.608657 0.801 0.423412
socrank4          1.629369  5.100653 0.460996  0.469211 3.473 0.000516 ***
socrank5          1.545115  4.688509 0.454658  0.457490 3.377 0.000732 ***
---
Signif. codes:  0 ‘***’ 0.001 ‘**’ 0.01 ‘*’ 0.05 ‘.’ 0.1 ‘ ’ 1

                 exp(coef) exp(-coef) lower .95 upper .95
sibdead5             1.586     0.6303    1.2262     2.053
sibdead5_10          1.183     0.8454    0.9225     1.517
sibdead10_15         1.176     0.8503    0.8894     1.555
famsize              1.089     0.9187    1.0331     1.147
dadD                 1.206     0.8291    0.9533     1.526
momD                 1.112     0.8992    0.8626     1.434
cohort               1.020     0.9805    1.0149     1.025
birth rank           1.015     0.9856    0.9624     1.070
socrank2             1.482     0.6749    0.4120     5.329
socrank3             1.628     0.6143    0.4938     5.367
socrank4             5.101     0.1961    2.0334    12.794
socrank5             4.689     0.2133    1.9126    11.493

Concordance= 0.717  (se = 0.015 )
Likelihood ratio test= 226.6  on 12 df,   p=<2e-16
Wald test            = 166.7  on 12 df,   p=<2e-16
Score (logrank) test = 203  on 12 df,   p=<2e-16,   Robust = 157.5  p=<2e-16

  (Note: the likelihood ratio and score tests assume independence of
     observations within a cluster, the Wald and robust score tests do not).
> cox.zph(coxmodel_cl4)
                   chisq df    p
sibdead5         0.49517  1 0.48
sibdead5_10      0.04690  1 0.83
sibdead10_15     0.56657  1 0.45
famsize          0.06827  1 0.79
dadD             0.51357  1 0.47
momD             0.00756  1 0.93
cohort           0.06723  1 0.80
birth rank       1.92192  1 0.17
socrank          2.70034  4 0.61
GLOBAL           7.01438 12 0.86
```

```
Call:
coxph(formula = Surv(start, end, dummy) ~ sibdead5 + sibdead5_10 + 
    sibdead10_15 + famsize + dadD + momD + cohort + birth rank + 
    socrank, data = sample2, method = "efron", cluster = idf)

  n= 41102, number of events= 203 

                       coef exp(coef)  se(coef) robust se      z Pr(>|z|)    
sibdead5           0.386772  1.472221  0.165635  0.176855  2.187  0.02875 *  
sibdead5_10        0.088385  1.092408  0.168763  0.168093  0.526  0.59902    
sibdead10_15       0.091466  1.095780  0.184185  0.191509  0.478  0.63293    
famsize           -0.009013  0.991027  0.039107  0.042707 -0.211  0.83285    
dadD              -0.072287  0.930264  0.152190  0.164362 -0.440  0.66008    
momD              -0.049084  0.952101  0.172919  0.189518 -0.259  0.79564    
cohort             0.017781  1.017940  0.003420  0.003807  4.671    3e-06 ***
birth rank         0.039015  1.039786  0.032872  0.033575  1.162  0.24522    
socrank2           0.639981  1.896444  0.913661  1.033326  0.619  0.53569    
socrank3           1.246246  3.477263  0.817154  0.830875  1.500  0.13364    
socrank4           2.126302  8.383808  0.721219  0.723216  2.940  0.00328 ** 
socrank5           1.990532  7.319429  0.716892  0.721912  2.757  0.00583 ** 
---
Signif. codes:  0 ‘***’ 0.001 ‘**’ 0.01 ‘*’ 0.05 ‘.’ 0.1 ‘ ’ 1

                  exp(coef) exp(-coef) lower .95 upper .95
sibdead5             1.4722     0.6792    1.0410     2.082
sibdead5_10          1.0924     0.9154    0.7858     1.519
sibdead10_15         1.0958     0.9126    0.7529     1.595
famsize              0.9910     1.0091    0.9115     1.078
dadD                 0.9303     1.0750    0.6741     1.284
momD                 0.9521     1.0503    0.6567     1.380
cohort               1.0179     0.9824    1.0104     1.026
birth rank           1.0398     0.9617    0.9736     1.111
socrank2             1.8964     0.5273    0.2502    14.372
socrank3             3.4773     0.2876    0.6823    17.721
socrank4             8.3838     0.1193    2.0316    34.597
socrank5             7.3194     0.1366    1.7782    30.128

Concordance= 0.701  (se = 0.022 )
Likelihood ratio test= 93.06  on 12 df,   p=1e-14
Wald test            = 56.11  on 12 df,   p=1e-07
Score (logrank) test = 75.94  on 12 df,   p=2e-11,   Robust = 74.84  p=4e-11

  (Note: the likelihood ratio and score tests assume independence of
     observations within a cluster, the Wald and robust score tests do not).
> cox.zph(coxmodel_cl4_S2)
                   chisq df     p
sibdead5          4.1121  1 0.043
sibdead5_10       0.0458  1 0.831
sibdead10_15      0.1902  1 0.663
famsize           0.1243  1 0.724
dadD              0.0440  1 0.834
momD              0.3393  1 0.560
cohort            1.2654  1 0.261
birth rank        5.6758  1 0.017
socrank           6.0395  4 0.196
GLOBAL           16.3453 12 0.176
```

```
Call:
coxph(formula = Surv(start, end, dummy) ~ sibdead5 + sibdead5_10 +
    sibdead10_15 + famsize + dadD + momD + cohort + birth rank +
    strata(idf), data = sample2, method = "efron")

  n= 41102, number of events= 203

                     coef exp(coef) se(coef)      z Pr(>|z|)
sibdead5          1.13874   3.12284  0.41584  2.738  0.00617 **
sibdead5_10       0.53830   1.71308  0.41107  1.309  0.19037
sibdead10_15      0.47774   1.61242  0.41175  1.160  0.24595
famsize          -0.14558   0.86452  0.37109 -0.392  0.69483
dadD             -0.27087   0.76272  0.53261 -0.509  0.61105
momD             -0.07831   0.92468  0.59176 -0.132  0.89472
cohort            0.01862   1.01880  0.04134  0.450  0.65235
birth rank        0.13694   1.14676  0.13490  1.015  0.31005
---
Signif. codes:  0 ‘***’ 0.001 ‘**’ 0.01 ‘*’ 0.05 ‘.’ 0.1 ‘ ’ 1

                  exp(coef) exp(-coef) lower .95 upper .95
sibdead5             3.1228     0.3202    1.3822     7.055
sibdead5_10          1.7131     0.5837    0.7654     3.834
sibdead10_15         1.6124     0.6202    0.7194     3.614
famsize              0.8645     1.1567    0.4177     1.789
dadD                 0.7627     1.3111    0.2685     2.166
momD                 0.9247     1.0815    0.2899     2.949
cohort               1.0188     0.9815    0.9395     1.105
birth rank           1.1468     0.8720    0.8803     1.494

Concordance= 0.62  (se = 0.054 )
Likelihood ratio test= 13.98  on 8 df,   p=0.08
Wald test            = 12.11  on 8 df,   p=0.1
Score (logrank) test = 13.27  on 8 df,   p=0.1

> cox.zph(coxmodel_fe4)
                    chisq df     p
sibdead5           0.5244  1 0.469
sibdead5_10        5.7067  1 0.017
sibdead10_15       6.4334  1 0.011
famsize            1.1293  1 0.288
dadD               0.0901  1 0.764
momD               0.0317  1 0.859
cohort             3.6359  1 0.057
birth rank         5.5619  1 0.018
GLOBAL            14.3799  8 0.072
```

A2-Full models for figure 3

```
Call:
coxph(formula = Surv(start, end, dummy) ~ sibdead5 + sibdead5_10 + 
    sibdead10_15 + famsize + dadD + momD + cohort + birth rank + 
    socrank, data = sample, method = "efron", cluster = idf)

  n= 75902, number of events= 8304 

                        coef  exp(coef)   se(coef)  robust se      z Pr(>|z|)    
sibdead5           0.0889959  1.0930762  0.0296907  0.0306592  2.903  0.00370 ** 
sibdead5_10        0.0556656  1.0572441  0.0281799  0.0291235  1.911  0.05596 .  
sibdead10_15       0.0460261  1.0471017  0.0309632  0.0312132  1.475  0.14033    
famsize           -0.0025546  0.9974486  0.0059498  0.0063271 -0.404  0.68639    
dadD               0.0756681  1.0786045  0.0241470  0.0261074  2.898  0.00375 ** 
momD               0.1431891  1.1539479  0.0255973  0.0270556  5.292 1.21e-07 ***
cohort             0.0012841  1.0012849  0.0003609  0.0003860  3.326  0.00088 ***
birth rank        -0.0122299  0.9878446  0.0060599  0.0062139 -1.968  0.04905 *  
socrank2          -0.0433941  0.9575339  0.0563588  0.0687967 -0.631  0.52820    
socrank3          -0.2166046  0.8052483  0.0587142  0.0702607 -3.083  0.00205 ** 
socrank4          -0.2636986  0.7682051  0.0454434  0.0543188 -4.855 1.21e-06 ***
socrank5          -0.3539909  0.7018813  0.0435255  0.0529750 -6.682 2.35e-11 ***
---
Signif. codes:  0 ‘***’ 0.001 ‘**’ 0.01 ‘*’ 0.05 ‘.’ 0.1 ‘ ’ 1

                  exp(coef) exp(-coef) lower .95 upper .95
sibdead5             1.0931     0.9148    1.0293    1.1608
sibdead5_10          1.0572     0.9459    0.9986    1.1193
sibdead10_15         1.0471     0.9550    0.9850    1.1132
famsize              0.9974     1.0026    0.9852    1.0099
dadD                 1.0786     0.9271    1.0248    1.1352
momD                 1.1539     0.8666    1.0944    1.2168
cohort               1.0013     0.9987    1.0005    1.0020
birth rank           0.9878     1.0123    0.9759    0.9999
socrank2             0.9575     1.0443    0.8367    1.0958
socrank3             0.8052     1.2419    0.7017    0.9241
socrank4             0.7682     1.3017    0.6906    0.8545
socrank5             0.7019     1.4247    0.6327    0.7787

Concordance= 0.549  (se = 0.004 )
Likelihood ratio test= 178.6  on 12 df,   p=<2e-16
Wald test            = 141.9  on 12 df,   p=<2e-16
Score (logrank) test = 186.5  on 12 df,   p=<2e-16,   Robust = 152.5  p=<2e-16

  (Note: the likelihood ratio and score tests assume independence of
     observations within a cluster, the Wald and robust score tests do not).
> cox.zph(coxmodel_cl4)
                     chisq df       p
sibdead5            4.5272  1   0.033
sibdead5_10         5.1079  1   0.024
sibdead10_15        0.0301  1   0.862
famsize             4.4041  1   0.036
dadD                3.5653  1   0.059
momD                4.6834  1   0.030
cohort             37.8837  1 7.5e-10
birth rank          0.2895  1   0.591
socrank            67.5179  4 7.6e-14
GLOBAL            110.0430 12 < 2e-16
```

```
Call:
coxph(formula = Surv(start, end, dummy) ~ sibdead5 + sibdead5_10 +
    sibdead10_15 + famsize + dadD + momD + cohort + birth rank +
    socrank, data = sample2, method = "efron", cluster = idf)

  n= 41102, number of events= 4104

                       coef  exp(coef)   se(coef)  robust se      z Pr(>|z|)
sibdead5          0.0125044  1.0125829  0.0400346  0.0407968  0.307 0.759221
sibdead5_10       0.0009663  1.0009667  0.0373203  0.0385534  0.025 0.980005
sibdead10_15      0.0422626  1.0431684  0.0405057  0.0413892  1.021 0.307206
famsize          -0.0157184  0.9844045  0.0088202  0.0100250 -1.568 0.116899
dadD              0.0330060  1.0335567  0.0335372  0.0376126  0.878 0.380201
momD              0.1210104  1.1286367  0.0362946  0.0399192  3.031 0.002434 **
cohort           -0.0002603  0.9997398  0.0005774  0.0006428 -0.405 0.685554
birth rank       -0.0124831  0.9875945  0.0075979  0.0076423 -1.633 0.102379
socrank2         -0.1758523  0.8387418  0.0747080  0.0875493 -2.009 0.044579 *
socrank3         -0.2417456  0.7852559  0.0765612  0.0915048 -2.642 0.008244 **
socrank4         -0.2483411  0.7800938  0.0585654  0.0677307 -3.667 0.000246 ***
socrank5         -0.3040157  0.7378492  0.0568414  0.0672259 -4.522 6.12e-06 ***
---
Signif. codes:  0 ‘***’ 0.001 ‘**’ 0.01 ‘*’ 0.05 ‘.’ 0.1 ‘ ’ 1

                 exp(coef) exp(-coef) lower .95 upper .95
sibdead5            1.0126     0.9876    0.9348    1.0969
sibdead5_10         1.0010     0.9990    0.9281    1.0795
sibdead10_15        1.0432     0.9586    0.9619    1.1313
famsize             0.9844     1.0158    0.9653    1.0039
dadD                1.0336     0.9675    0.9601    1.1126
momD                1.1286     0.8860    1.0437    1.2205
cohort              0.9997     1.0003    0.9985    1.0010
birth rank          0.9876     1.0126    0.9729    1.0025
socrank2            0.8387     1.1923    0.7065    0.9958
socrank3            0.7853     1.2735    0.6563    0.9395
socrank4            0.7801     1.2819    0.6831    0.8908
socrank5            0.7378     1.3553    0.6468    0.8418

Concordance= 0.537  (se = 0.006 )
Likelihood ratio test= 53.52  on 12 df,   p=3e-07
Wald test            = 45.24  on 12 df,   p=9e-06
Score (logrank) test = 55.68  on 12 df,   p=1e-07,   Robust = 45.06  p=1e-05

  (Note: the likelihood ratio and score tests assume independence of
     observations within a cluster, the Wald and robust score tests do not).
> cox.zph(coxmodel_cl4_S2)
                   chisq df       p
sibdead5          2.2800  1 0.13106
sibdead5_10       2.6416  1 0.10410
sibdead10_15      1.0129  1 0.31420
famsize           7.1596  1 0.00746
dadD              0.5336  1 0.46510
momD              2.8180  1 0.09321
cohort           14.8354  1 0.00012
birth rank        0.0228  1 0.87993
socrank          22.6336  4 0.00015
GLOBAL           46.9865 12 4.7e-06
```

```
Call:
coxph(formula = Surv(start, end, dummy) ~ sibdead5 + sibdead5_10 + 
    sibdead10_15 + famsize + dadD + momD + cohort + birth rank + 
    strata(idf), data = sample2, method = "efron")

  n= 41102, number of events= 4104 

                       coef exp(coef)  se(coef)      z Pr(>|z|)  
sibdead5           0.043200  1.044146  0.078863  0.548    0.584  
sibdead5_10       -0.057072  0.944526  0.081598 -0.699    0.484  
sibdead10_15      -0.047344  0.953759  0.084859 -0.558    0.577  
famsize           -0.012963  0.987121  0.081260 -0.160    0.873  
dadD               0.231750  1.260804  0.112274  2.064    0.039 *
momD               0.120991  1.128615  0.130524  0.927    0.354  
cohort             0.007920  1.007951  0.008112  0.976    0.329  
birth rank        -0.039983  0.960806  0.026156 -1.529    0.126  
---
Signif. codes:  0 ‘***’ 0.001 ‘**’ 0.01 ‘*’ 0.05 ‘.’ 0.1 ‘ ’ 1

                  exp(coef) exp(-coef) lower .95 upper .95
sibdead5             1.0441     0.9577    0.8946     1.219
sibdead5_10          0.9445     1.0587    0.8049     1.108
sibdead10_15         0.9538     1.0485    0.8076     1.126
famsize              0.9871     1.0130    0.8418     1.158
dadD                 1.2608     0.7931    1.0118     1.571
momD                 1.1286     0.8860    0.8739     1.458
cohort               1.0080     0.9921    0.9921     1.024
birth rank           0.9608     1.0408    0.9128     1.011

Concordance= 0.511  (se = 0.013 )
Likelihood ratio test= 7.94  on 8 df,   p=0.4
Wald test            = 7.9  on 8 df,   p=0.4
Score (logrank) test = 7.93  on 8 df,   p=0.4

> cox.zph(coxmodel_fe4)
                  chisq df    p
sibdead5          0.822  1 0.36
sibdead5_10       0.876  1 0.35
sibdead10_15      0.509  1 0.48
famsize           3.531  1 0.06
dadD              1.024  1 0.31
momD              0.703  1 0.40
cohort            0.701  1 0.40
birth rank        0.409  1 0.52
GLOBAL            9.114  8 0.33
```

A3-Number of experienced sibling death(s)

```
Call:
coxph(formula = Surv(start, end, dummy) ~ sibdead5 + sibdead5_10 +
    sibdead10_15 + famsize + dadD + momD + cohort + birth rank +
    socrank, data = sample, method = "efron", cluster = idf)

  n= 75902, number of events= 379

                      coef exp(coef) se(coef) robust se     z Pr(>|z|)
sibdead5          0.374332  1.454019 0.098797  0.101825 3.676 0.000237 ***
sibdead5_10       0.191595  1.211180 0.092567  0.097244 1.970 0.048810 *
sibdead10_15      0.144705  1.155699 0.105869  0.110882 1.305 0.191879
famsize           0.086169  1.089990 0.025769  0.026374 3.267 0.001086 **
dadD              0.193350  1.213307 0.110598  0.119221 1.622 0.104852
momD              0.110195  1.116496 0.121324  0.129165 0.853 0.393587
cohort            0.019950  1.020150 0.002249  0.002489 8.014 1.11e-15 ***
birth rank        0.013428  1.013518 0.026133  0.026811 0.501 0.616486
socrank2          0.397187  1.487634 0.605988  0.653648 0.608 0.543422
socrank3          0.513969  1.671913 0.586173  0.611074 0.841 0.400298
socrank4          1.649980  5.206874 0.461126  0.470090 3.510 0.000448 ***
socrank5          1.564472  4.780150 0.454905  0.458573 3.412 0.000646 ***
---
Signif. codes:  0 ‘***’ 0.001 ‘**’ 0.01 ‘*’ 0.05 ‘.’ 0.1 ‘ ’ 1

                  exp(coef) exp(-coef) lower .95 upper .95
sibdead5              1.454     0.6877    1.1910     1.775
sibdead5_10           1.211     0.8256    1.0010     1.465
sibdead10_15          1.156     0.8653    0.9300     1.436
famsize               1.090     0.9174    1.0351     1.148
dadD                  1.213     0.8242    0.9605     1.533
momD                  1.116     0.8957    0.8668     1.438
cohort                1.020     0.9802    1.0152     1.025
birth rank            1.014     0.9867    0.9616     1.068
socrank2              1.488     0.6722    0.4132     5.357
socrank3              1.672     0.5981    0.5047     5.538
socrank4              5.207     0.1921    2.0722    13.083
socrank5              4.780     0.2092    1.9458    11.743

Concordance= 0.718  (se = 0.015 )
Likelihood ratio test= 230.4  on 12 df,   p=<2e-16
Wald test            = 167.6  on 12 df,   p=<2e-16
Score (logrank) test = 205.8  on 12 df,   p=<2e-16,   Robust = 157.8  p=<2e-16

  (Note: the likelihood ratio and score tests assume independence of
     observations within a cluster, the Wald and robust score tests do not).
> cox.zph(coxmodel_cl4)
                   chisq df    p
sibdead5          0.4437  1 0.51
sibdead5_10       0.3448  1 0.56
sibdead10_15      1.0206  1 0.31
famsize           0.0571  1 0.81
dadD              0.4722  1 0.49
momD              0.0139  1 0.91
cohort            0.0804  1 0.78
birth rank        1.8379  1 0.18
socrank           2.7177  4 0.61
GLOBAL            7.7343 12 0.81
```

```
Call:
coxph(formula = Surv(start, end, dummy) ~ sibdead5 + sibdead5_10 + 
    sibdead10_15 + famsize + dadD + momD + cohort + birth rank + 
    socrank, data = sample2, method = "efron", cluster = idf)

  n= 41102, number of events= 203 

                       coef exp(coef)  se(coef) robust se      z Pr(>|z|)    
sibdead5           0.362837  1.437401  0.131007  0.140118  2.590  0.00961 ** 
sibdead5_10        0.058686  1.060443  0.127769  0.125155  0.469  0.63914    
sibdead10_15       0.148106  1.159636  0.136879  0.150795  0.982  0.32601    
famsize           -0.007887  0.992144  0.039147  0.042603 -0.185  0.85313    
dadD              -0.064310  0.937714  0.152036  0.163183 -0.394  0.69351    
momD              -0.047870  0.953257  0.172806  0.189060 -0.253  0.80011    
cohort             0.018122  1.018287  0.003440  0.003804  4.764  1.9e-06 ***
birth rank         0.037362  1.038068  0.033006  0.033297  1.122  0.26184    
socrank2           0.663808  1.942175  0.913735  1.036904  0.640  0.52205    
socrank3           1.295453  3.652650  0.817756  0.836097  1.549  0.12128    
socrank4           2.163487  8.701431  0.721371  0.725961  2.980  0.00288 ** 
socrank5           2.017942  7.522830  0.717140  0.725185  2.783  0.00539 ** 
---
Signif. codes:  0 ‘***’ 0.001 ‘**’ 0.01 ‘*’ 0.05 ‘.’ 0.1 ‘ ’ 1

                  exp(coef) exp(-coef) lower .95 upper .95
sibdead5             1.4374     0.6957    1.0922     1.892
sibdead5_10          1.0604     0.9430    0.8298     1.355
sibdead10_15         1.1596     0.8623    0.8629     1.558
famsize              0.9921     1.0079    0.9127     1.079
dadD                 0.9377     1.0664    0.6810     1.291
momD                 0.9533     1.0490    0.6581     1.381
cohort               1.0183     0.9820    1.0107     1.026
birth rank           1.0381     0.9633    0.9725     1.108
socrank2             1.9422     0.5149    0.2545    14.822
socrank3             3.6526     0.2738    0.7094    18.806
socrank4             8.7014     0.1149    2.0973    36.102
socrank5             7.5228     0.1329    1.8159    31.164

Concordance= 0.701  (se = 0.022 )
Likelihood ratio test= 96.02  on 12 df,   p=3e-15
Wald test            = 57.9  on 12 df,   p=5e-08
Score (logrank) test = 78.71  on 12 df,   p=7e-12,   Robust = 74.98  p=4e-11

  (Note: the likelihood ratio and score tests assume independence of
     observations within a cluster, the Wald and robust score tests do not).
> cox.zph(coxmodel_cl4_S2)
                    chisq df     p
sibdead5           3.3910  1 0.066
sibdead5_10        0.0922  1 0.761
sibdead10_15       0.8097  1 0.368
famsize            0.1184  1 0.731
dadD               0.0391  1 0.843
momD               0.3052  1 0.581
cohort             1.2890  1 0.256
birth rank         5.7587  1 0.016
socrank            6.0004  4 0.199
GLOBAL            16.1131 12 0.186
```

```
Call:
coxph(formula = Surv(start, end, dummy) ~ sibdead5 + sibdead5_10 +
    sibdead10_15 + famsize + dadD + momD + cohort + birth rank +
    strata(idf), data = sample2, method = "efron")

  n= 41102, number of events= 203

                      coef exp(coef) se(coef)      z Pr(>|z|)
sibdead5           0.80389   2.23421  0.32548  2.470   0.0135 *
sibdead5_10        0.42510   1.52975  0.33785  1.258   0.2083
sibdead10_15       0.24077   1.27222  0.29634  0.812   0.4165
famsize           -0.16405   0.84870  0.37388 -0.439   0.6608
dadD              -0.18343   0.83241  0.52527 -0.349   0.7269
momD              -0.04375   0.95719  0.58832 -0.074   0.9407
cohort             0.02774   1.02812  0.04128  0.672   0.5017
birth rank         0.08889   1.09296  0.13654  0.651   0.5150
---
Signif. codes:  0 ‘***’ 0.001 ‘**’ 0.01 ‘*’ 0.05 ‘.’ 0.1 ‘ ’ 1

                  exp(coef) exp(-coef) lower .95 upper .95
sibdead5             2.2342     0.4476    1.1805     4.228
sibdead5_10          1.5297     0.6537    0.7889     2.966
sibdead10_15         1.2722     0.7860    0.7117     2.274
famsize              0.8487     1.1783    0.4079     1.766
dadD                 0.8324     1.2013    0.2973     2.330
momD                 0.9572     1.0447    0.3021     3.032
cohort               1.0281     0.9726    0.9482     1.115
birth rank           1.0930     0.9149    0.8363     1.428

Concordance= 0.607  (se = 0.055 )
Likelihood ratio test= 11.71  on 8 df,   p=0.2
Wald test            = 10.37  on 8 df,   p=0.2
Score (logrank) test = 11.31  on 8 df,   p=0.2

> cox.zph(coxmodel_fe4)
                    chisq df      p
sibdead5           0.0560  1 0.8129
sibdead5_10        3.8429  1 0.0500
sibdead10_15       7.1126  1 0.0077
famsize            1.1260  1 0.2886
dadD               0.1207  1 0.7282
momD               0.0274  1 0.8686
cohort             3.3737  1 0.0662
birth rank         5.0517  1 0.0246
GLOBAL            11.1298  8 0.1945
```

A4- Raw effects of experienced sibling death(s)

```
Call:
coxph(formula = Surv(start, end, dummy) ~ sibdead5 + sibdead5_10 +
    sibdead10_15, data = sample, method = "efron", cluster = idf)

  n= 75902, number of events= 379

                   coef exp(coef) se(coef) robust se     z Pr(>|z|)
sibdead5         0.4603    1.5846   0.1229    0.1268 3.630 0.000283 ***
sibdead5_10      0.1338    1.1432   0.1270    0.1274 1.051 0.293470
sibdead10_15     0.1686    1.1836   0.1367    0.1398 1.206 0.227856
---
Signif. codes:  0 ‘***’ 0.001 ‘**’ 0.01 ‘*’ 0.05 ‘.’ 0.1 ‘ ’ 1

                 exp(coef) exp(-coef) lower .95 upper .95
sibdead5             1.585     0.6311    1.2359     2.032
sibdead5_10          1.143     0.8747    0.8906     1.467
sibdead10_15         1.184     0.8449    0.9000     1.557

Concordance= 0.551  (se = 0.017 )
Likelihood ratio test= 17.6  on 3 df,   p=5e-04
Wald test            = 17.81  on 3 df,   p=5e-04
Score (logrank) test = 19.53  on 3 df,   p=2e-04,   Robust = 13.31  p=0.004

  (Note: the likelihood ratio and score tests assume independence of
     observations within a cluster, the Wald and robust score tests do not).
> cox.zph(coxmodel_cl_raw)
                   chisq df    p
sibdead5         0.10866  1 0.74
sibdead5_10      0.00594  1 0.94
sibdead10_15     1.79527  1 0.18
GLOBAL           2.10667  3 0.55
```

```
Call:
coxph(formula = Surv(start, end, dummy) ~ sibdead5 + sibdead5_10 + 
    sibdead10_15, data = sample2, method = "efron", cluster = idf)

  n= 41102, number of events= 203 

                 coef exp(coef) se(coef) robust se      z Pr(>|z|)  
sibdead5      0.34847   1.41689  0.16264   0.17099  2.038   0.0416 *
sibdead5_10  -0.02379   0.97649  0.16837   0.16938 -0.140   0.8883  
sibdead10_15 -0.02462   0.97568  0.18333   0.19030 -0.129   0.8971  
---
Signif. codes:  0 ‘***’ 0.001 ‘**’ 0.01 ‘*’ 0.05 ‘.’ 0.1 ‘ ’ 1

             exp(coef) exp(-coef) lower .95 upper .95
sibdead5        1.4169     0.7058    1.0134     1.981
sibdead5_10     0.9765     1.0241    0.7006     1.361
sibdead10_15    0.9757     1.0249    0.6719     1.417

Concordance= 0.567  (se = 0.023 )
Likelihood ratio test= 4.34  on 3 df,   p=0.2
Wald test            = 4.23  on 3 df,   p=0.2
Score (logrank) test = 4.65  on 3 df,   p=0.2,   Robust = 3.46  p=0.3

  (Note: the likelihood ratio and score tests assume independence of
     observations within a cluster, the Wald and robust score tests do not).
> cox.zph(coxmodel_cl_S2_raw)
             chisq df     p
sibdead5     3.405  1 0.065
sibdead5_10  0.037  1 0.848
sibdead10_15 0.538  1 0.463
GLOBAL       4.431  3 0.218
```

```
Call:
coxph(formula = Surv(start, end, dummy) ~ sibdead5 + sibdead5_10 +
    sibdead10_15 + strata(idf), data = sample2, method = "efron")

  n= 41102, number of events= 203

                    coef exp(coef) se(coef)     z Pr(>|z|)
sibdead5          0.8829    2.4179   0.3903 2.262   0.0237 *
sibdead5_10       0.1439    1.1548   0.3538 0.407   0.6841
sibdead10_15      0.1466    1.1579   0.3637 0.403   0.6869
---
Signif. codes:  0 ‘***’ 0.001 ‘**’ 0.01 ‘*’ 0.05 ‘.’ 0.1 ‘ ’ 1

                  exp(coef) exp(-coef) lower .95 upper .95
sibdead5              2.418     0.4136    1.1252     5.196
sibdead5_10           1.155     0.8660    0.5773     2.310
sibdead10_15          1.158     0.8636    0.5677     2.362

Concordance= 0.577  (se = 0.04 )
Likelihood ratio test= 5.81  on 3 df,   p=0.1
Wald test            = 5.4  on 3 df,   p=0.1
Score (logrank) test = 5.68  on 3 df,   p=0.1

> cox.zph(coxmodel_fe_raw)
                  chisq df     p
sibdead5          0.355  1 0.552
sibdead5_10       3.086  1 0.079
sibdead10_15      5.032  1 0.025
GLOBAL            6.553  3 0.088
```

A5-Sensitivity Window 1

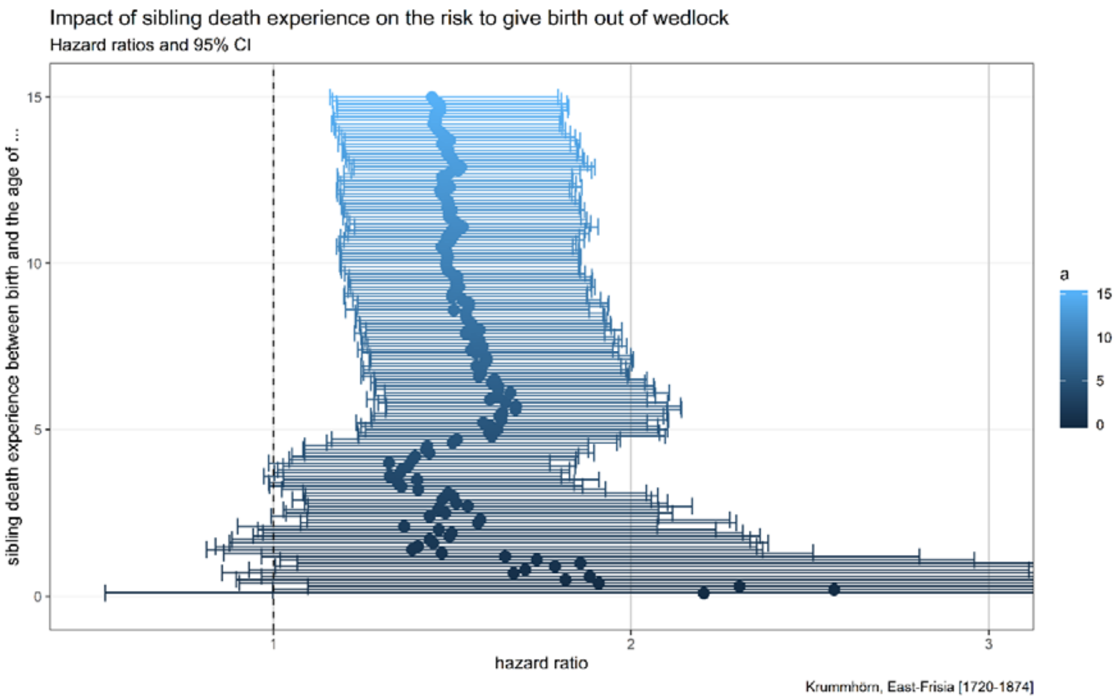

A6-Sensitivity Window 2

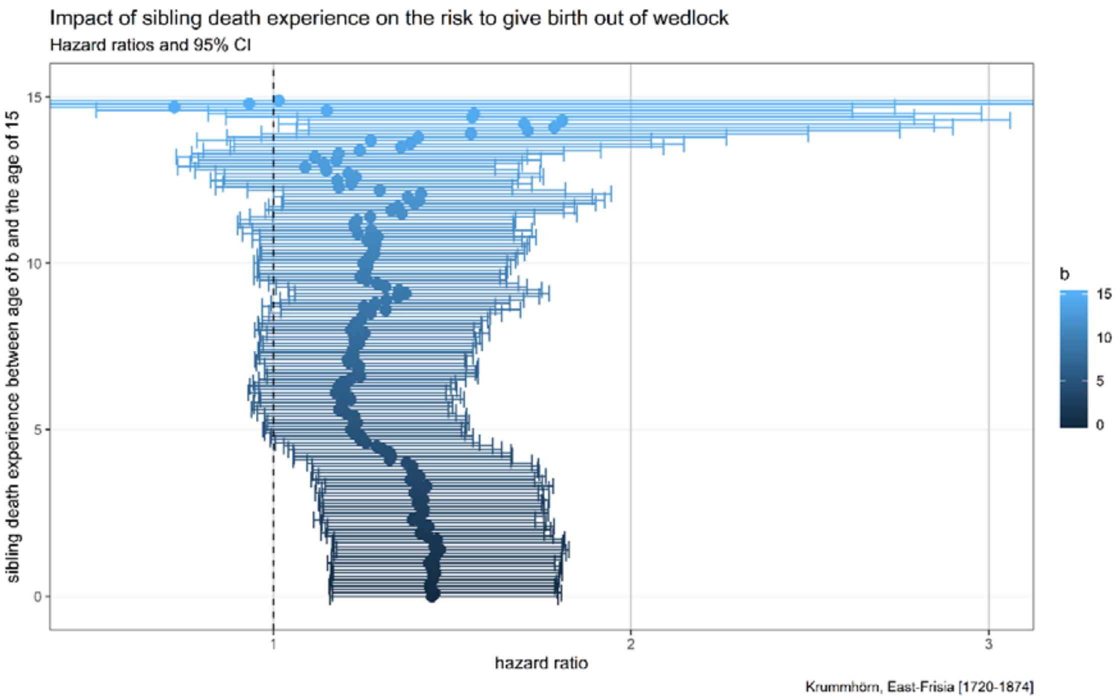

Supplement: Supplementary file 1 — (PDF 262 kb) [file 12110_2020_9368_MOESM1_ESM.pdf]
